# Sialylation of *Campylobacter jejuni* Lipo-Oligosaccharides: Impact on Phagocytosis and Cytokine Production in Mice

**DOI:** 10.1371/journal.pone.0034416

**Published:** 2012-03-28

**Authors:** Ruth Huizinga, Alistair S. Easton, Anne M. Donachie, Jim Guthrie, Wouter van Rijs, Astrid Heikema, Louis Boon, Janneke N. Samsom, Bart C. Jacobs, Hugh J. Willison, Carl S. Goodyear

**Affiliations:** 1 Department of Immunology, Erasmus MC, University Medical Center Rotterdam, Rotterdam, The Netherlands; 2 Institute of Infection, Immunity and Inflammation, College of Medical, Veterinary and Life Sciences, University of Glasgow, Glasgow, United Kingdom; 3 Department of Bacteriology, Southern General Hospital, Glasgow, United Kingdom; 4 Department of Neurology, Erasmus MC, University Medical Center Rotterdam, Rotterdam, The Netherlands; 5 Department of Microbiology, Erasmus MC, University Medical Center Rotterdam, Rotterdam, The Netherlands; 6 Bioceros BV, Utrecht, The Netherlands; 7 Division Gastroenterology and Nutrition, Department of Paediatrics, Erasmus MC, University Medical Center Rotterdam, Rotterdam, The Netherlands; Oklahoma Medical Research Foundation, United States of America

## Abstract

**Background:**

Guillain-Barré syndrome (GBS) is a post-infectious polyradiculoneuropathy, frequently associated with antecedent *Campylobacter jejuni* (*C. jejuni*) infection. The presence of sialic acid on *C. jejuni* lipo-oligosaccharide (LOS) is considered a risk factor for development of GBS as it crucially determines the structural homology between LOS and gangliosides, explaining the induction of cross-reactive neurotoxic antibodies. Sialylated *C. jejuni* are recognised by TLR4 and sialoadhesin; however, the functional implications of these interactions *in vivo* are unknown.

**Methodology/Principal Findings:**

In this study we investigated the effects of bacterial sialylation on phagocytosis and cytokine secretion by mouse myeloid cells *in vitro* and *in vivo*. Using fluorescently labelled GM1a/GD1a ganglioside-mimicking *C. jejuni* strains and corresponding (Cst-II-mutant) control strains lacking sialic acid, we show that sialylated *C. jejuni* was more efficiently phagocytosed *in vitro* by BM-MΦ, but not by BM-DC. In addition, LOS sialylation increased the production of IL-10, IL-6 and IFN-β by both BM-MΦ and BM-DC. Subsequent *in vivo* experiments revealed that sialylation augmented the deposition of fluorescent bacteria in splenic DC, but not macrophages. In addition, sialylation significantly amplified the production of type I interferons, which was independent of pDC.

**Conclusions/Significance:**

These results identify novel immune stimulatory effects of *C. jejuni* sialylation, which may be important in inducing cross-reactive humoral responses that cause GBS.

## Introduction

*C. jejuni* is one of the most common causes of bacterial gastroenteritis affecting approximately 50 per 100,000 individuals each year in Europe [1]. In most cases, gastroenteritis resolves within a week, however, a minority of infected individuals develop post-infectious GBS. This is a life-threatening neurological disease in which peripheral nerves including spinal roots are damaged (polyradiculoneuropathy), leading to a rapid and ascending paralysis of the extremities. Patients with a preceding *C. jejuni* infection have a more severe form of GBS with poorer outcome as compared to patients without preceding *C. jejuni* infection [2].

Nerve damage that precipitates *C. jejuni*-related GBS is the result of antibodies that are generated against sialylated LOS present on *C. jejuni* [2]. These antibodies cross-react with structurally similar glycolipids present on peripheral nerve membranes, and initiate complement activation and neuronal degeneration [3], [4]. Animal studies have demonstrated that *C. jejuni* LOS induces ganglioside-reactive antibodies that lead to subsequent GBS-like paralysis [5], [6].

*C. jejuni* strains isolated from GBS patients more frequently express the enzyme *Campylobacter* sialic acid transferase-II (Cst-II), which is responsible for the sialylation of LOS [7]. The sialic acid residue is required for the structural homology between LOS and gangliosides like GM1a and GD1a and crucially determines antibody cross-reactivity [8]. Only a limited number of individuals develop GBS after infection with *C. jejuni* and it is likely that susceptibility is determined by both pathogen and host factors.

Sialylation is known to be beneficial to many pathogens. In the case of *C. jejuni* it increases epithelial invasiveness [9] and protects against complement-mediated lysis [10]. In a possible example of the “Red Queen effect”, which symbolises the continuous evolutionary battle between species, the mammalian immune system has evolved sialic acid specific receptors, such as the rapidly evolving Siglec family (sialic acid-binding immunoglobulin-like lectins) [11]. While interaction of sialylated pathogens with Siglecs typically results in reduced immune responses due to the presence of ITIMs in many of the Siglecs, in the case of *C. jejuni* sialylation is associated with increased host immune responses. For example, in a cohort of patients with *C. jejuni* enteritis, sialylation was associated with higher levels of specific IgM antibodies and an increased severity of gastroenteritis, which may be the result of a more robust immune response [12]. The specific role of sialylated LOS in enhanced inflammatory responses was demonstrated recently by *in vitro* studies showing that sialylated LOS more efficiently stimulates human DC through TLR4, thus amplifying DC-mediated B-cell proliferation [13]. Similarly, sialylated pathogens may be more prone to scavenging by specialised macrophages, as exemplified by binding of sialylated *C. jejuni* to sialoadhesin, also known as Siglec-1 [14].

In the far majority of GBS patients, loss of self-tolerance is transient. The mechanisms that drive this loss of tolerance and subsequent re-establishment of tolerance are poorly understood. Likewise, the precise mechanism by which sialylation affects the immunogenicity of *C. jejuni in vivo* remains unknown. Given the above evidence, it is likely that sialylation does not only supply the requisite molecular mimicry to break tolerance, but also directly drives more robust inflammatory responses which, in susceptible individuals, may allow autoreactive B cell clones to bypass the tolerance mechanisms.

We hypothesize that the selective binding of sialylated *C. jejuni* to innate receptors like Siglecs and TLR4 results in targeting to myeloid cell populations with high expression of these receptors such as splenic metallophilic macrophages. These initial events may alter signalling and crucially determine subsequent immune responses by influencing processes like antigen presentation [15], regulatory T cell expansion [16] and B-cell activation [17].

In this study we investigated the functional role of LOS sialylation on cytokine responses and phagocytosis of *C. jejuni* by murine myeloid lineage antigen presenting cells, macrophages and DC. We show that the presence of sialic acids on *C. jejuni* promotes enhanced phagocytosis *in vitro* by BM-MΦ but not BM-DC. Furthermore, sialic acid on *C. jejuni* LOS results in increased cytokine responses by both myeloid cell populations. The increased phagocytosis by BM-MΦ did not translate into a preferential uptake of sialylated *C. jejuni* by specialised macrophage populations in the spleen *in vivo*. However a small increase was noted in the number of splenic DC that ingested sialylated bacteria. These DC may account for the selective and robust increase in type I interferon production in response to sialylated *C. jejuni*.

## Results

### BM-MΦ, but not BM-DC, show increased phagocytosis of sialylated *C. jejuni*

To investigate the impact of *C. jejuni* sialylation on functional responses by myeloid cells, we used different isogenic *C. jejuni* strains. The GB2 and GB11 wildtype strains express LOS that mimic gangliosides GM1a and GD1a, whereas the GB2 and GB11 Cst-II mutant strains do not mimic gangliosides. All strains were fluorescently labelled using CFSE in order to facilitate tracking during phagocytosis. To ensure comparable fluorescence intensities for the quantitative phagocytosis studies, we performed flow cytometry after the CFSE-labelling. Figure S1 shows that the mean fluorescence intensities of wildtype and Cst-II mutant strains were indeed comparable (MFI 57.1 vs. 57.6 for GB2 wt and mutant respectively, and MFI 83.7 vs. 81.2 for GB11 wt and mutant respectively). The *C. jejuni* were heat-inactivated to prevent dilution through bacterial cell division.

As sialylation has previously been shown to enhance the rate of phagocytosis of *Neisseria meningitides* [18], we hypothesised that sialylation of *C. jejuni* would result in increased phagocytosis by myeloid cells. Labelled GB11 bacteria were added to BM-MΦ or BM-DC and the fluorescence intensity was determined as a measure of bacterial phagocytosis.

**Figure 1:**
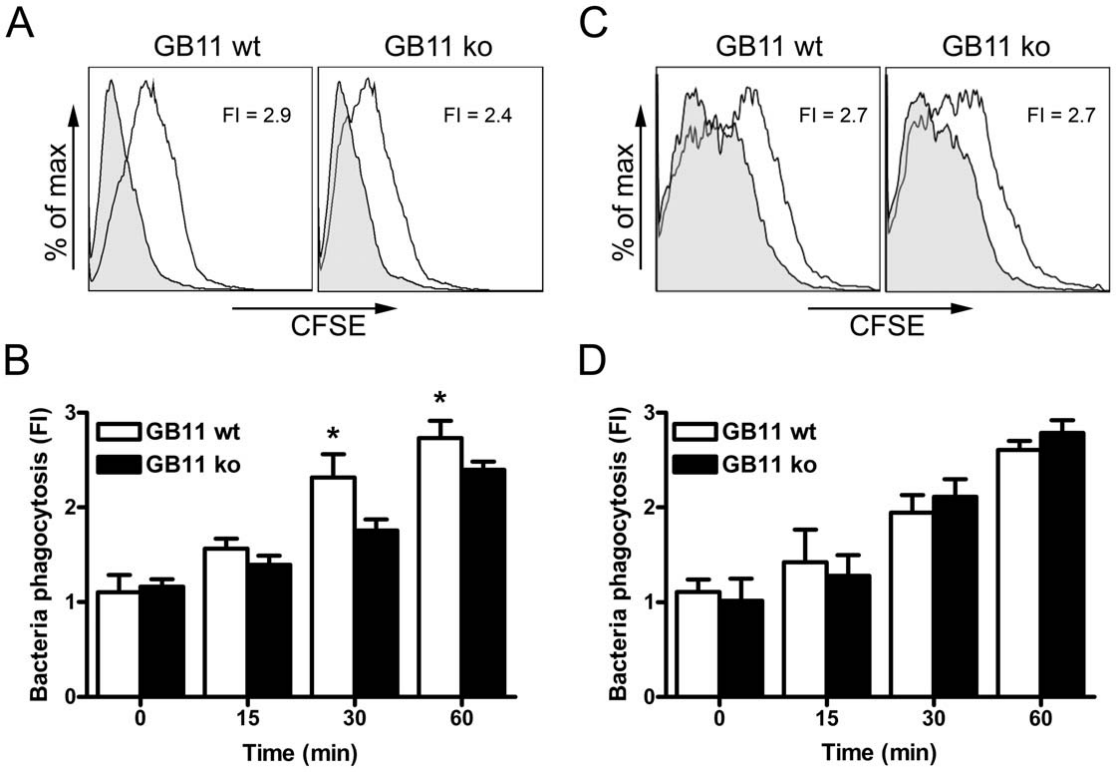
Increased phagocytosis of sialylated *C. jejuni* by BM-MΦ. (A) BM-MΦ or (C) BM-DC were incubated with CFSE-labelled wt or Cst-II mutant GB11 for 60 min at 37°C (open histograms) or 4°C (filled histograms). Phagocytosis of *C. jejuni* by F4/80+ cells or CD11c^+^ cells was quantified as fold increase in fluorescence intensity using background fluorescence of 4°C, NaN_3_ cultured cells as a reference. Phagocytosis of sialylated wt GB11 and unsialylated GB11 for 0, 15, 30 and 60 min shows that sialylation increases *C. jejuni* phagocytosis by BM-MΦ (B) but not DM-DC (D). One representative experiment out of 3 is shown (B and D; means ± sd of triplicates). * p<0.05; t-test.

BM-MΦ incubated with sialylated GB11 wt showed higher fluorescence intensities than BM-MΦ incubated with Cst-II mutant GB11 (Figure 1A and B). The difference was most pronounced after 30 min of incubation; the fold increase of cells incubated with GB11 wt was 2.3±0.2 compared to 1.8±0.1 when cells were incubated with GB11 Cst-II mutant bacteria (p<0.05; t test). The intracellular location of the wildtype and knockout bacteria was confirmed by microscopy (Figure S2). In contrast, BM-DC showed no difference in fluorescence intensity between cells incubated with GB11 wt and GB11 Cst-II mutant bacteria (Figure 1C and D).

Although it remains to be determined, it is possible that the sialylated epitope phagocytic receptor is a Siglec. Interestingly, sialoadhesin, which binds to α2,3-linked sialic acids [13], could facilitate the enhanced phagocytosis identified in BM-MΦ. In support of this concept, Facs analysis demonstrated high expression of sialoadhesin on BM-MΦ but not BM-DC. In addition, sialoadhesin was upregulated on BM-MΦ but not on BM-DC, following stimulation with *C. jejuni* LOS (Figure S3).

### LOS sialylation enhances myeloid cell cytokine production

Given the interaction of LOS with TLR4 and the potential interaction of sialylated epitopes with Siglecs we investigated the influence of *C. jejuni* sialylation on cytokine responses in myeloid-derived antigen presenting cells. As other TLR/NLR ligands present in *C. jejuni* may have confounding effects, we stimulated BM-MΦ and BM-DC with a titrating dose of purified LOS derived from GB11 wt and GB11 Cst-II mutant strains and measured cytokine responses in the culture supernatant.

**Figure 2:**
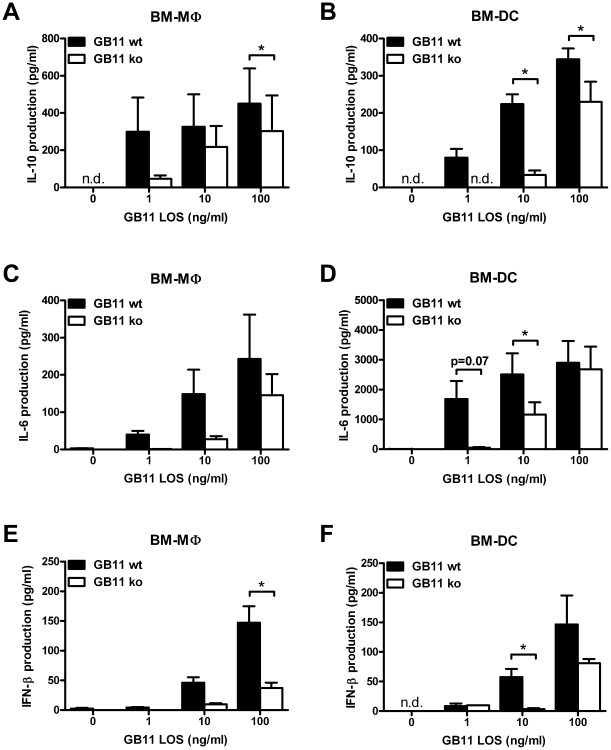
Sialylated GB11 LOS induces higher cytokine levels by myeloid cells. BM-MΦ (A, C and E) and BM-DC (B, D and F) were incubated overnight (for IL-6 and IL-10) or for 4 h (IFN-β) with purified LOS derived from GB11 *C. jejuni* or Cst-II mutant *C. jejuni* and cytokine production was measured by ELISA. Sialylated LOS induces more IL-10 (A and B), IL-6 (C and D) and IFN-β (E and F) than non-sialylated LOS. Graphs represent pooled data as mean ± standard error from 3 (IL-10 and IFN-β) or 4 (IL-6) independent experiments. * p<0.05; paired t-test, n.d.: not detectable.

Overnight stimulation with *C. jejuni* LOS induced a strong dose-dependent IL-6 and IL-10 response in BM-DC and BM-MΦ. However, myeloid cells stimulated with GB11 wt LOS produced significantly higher levels of IL-10 and IL-6 than cells stimulated with Cst-II mutant GB11 derived LOS (p<0.05; paired t test; Figure 2). This difference was observed using different concentrations of LOS, starting as low as 1 ng/ml. Similarly, the production of IFN-β, which is induced by *C. jejuni* following MyD88-independent TLR4 activation [19], was significantly higher in both BM-DC and BM-MΦ after 4 h stimulation with sialylated LOS (Figure 2E and F). The importance of sialylation was further studied using LOS derived from the additional sialylated and non-sialylated isogenic *C. jejuni* strains GB2 wt and ko. BM-DC and BM-MΦ stimulated with sialylated GB2 produced significantly higher levels of IL-6 and IL-10, like GB11 wt, when compared to the non-sialylated counterpart (data not shown). These results confirm that the increased cytokine responsiveness is dependent on the presence of a sialylated epitope.

To confirm that LOS induced BM-DC IL-10 production was TLR4 dependent and that contamination was not present. BM-DC derived from the C3H/HeJ mouse strain (Tlr4 mutant, LPS hyporesponsive) and the control C3H/HeN strain (LPS responsive) were stimulated as before and IL-10 assayed. No IL-10 was produced in the absence of TLR4 signalling (data not shown). Thus confirming that following LOS stimulation, the sialylated epitope enhanced IL-10 production is TLR4-dependent and that contamination by other TLR/NLR ligands is highly unlikely in our preparations.

### *C. jejuni* is scavenged by macrophages in the splenic marginal zone

Having demonstrated sialylation specific differences in macrophage phagocytosis *in vitro*, we subsequently investigated whether *in vivo* phagocytosis of *C. jejuni* was also affected by sialylation. Because sialylation also increases invasion and translocation over gut epithelial tissues, potentially leading to unequal numbers of bacteria in the blood stream, we injected CFSE-labelled GB2 wt or Cst-II mutant bacteria intravenously into C57BL/6 mice and isolated the spleens after various time points to assess *C. jejuni* localisation.

**Figure 3:**
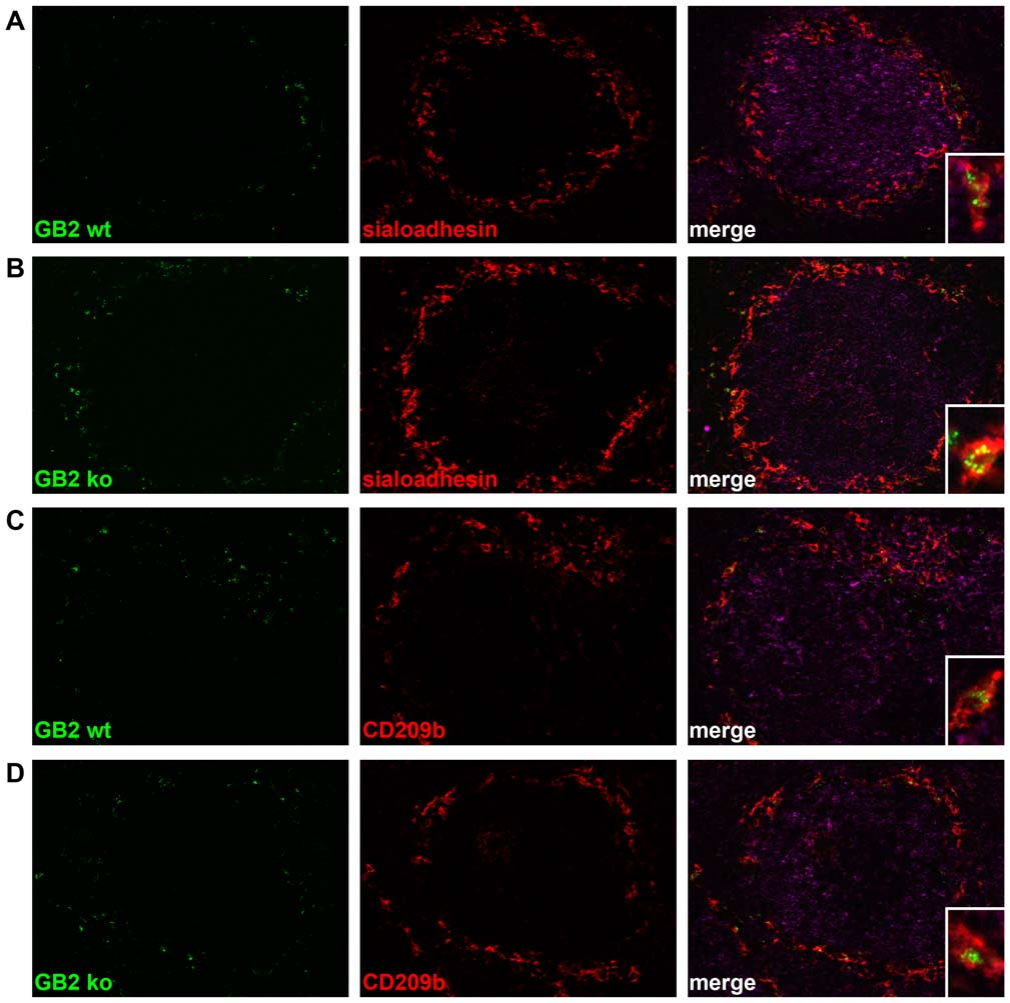
Both sialylated and non-sialylated *C. jejuni* are scavenged by macrophages in the splenic marginal zone. Mice were injected with CFSE-labelled GB2 wt (A and C) or Cst-II mutant (B) bacteria and spleens were isolated after 1 h. Spleen sections were stained for sialoadhesin (A and B) or CD209b (C and D) and counterstained for B220 (purple). Insets show colocalisation of GB2 wt and ko bacteria with sialoadhesin and CD209b positive macrophages.

**Figure 4:**
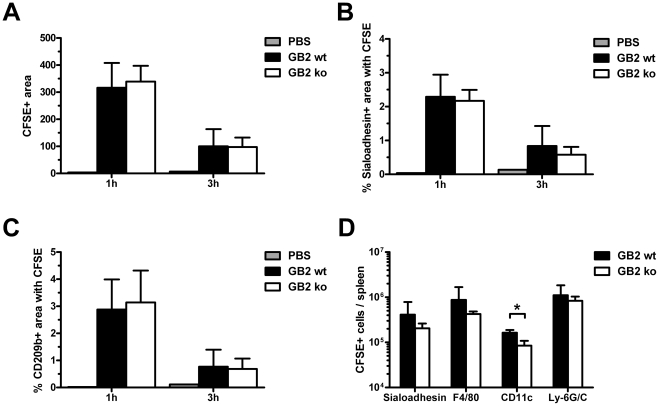
Sialylation of *C. jejuni* increases *in vivo* phagocytosis by splenic DC but not macrophages. Mice were sacrificed 1 h or 3 h after i.v. injection of PBS or CFSE-labelled GB2 (wt or Cst-II mutant). Bacterial deposition in spleen sections, stained for sialoadhesin, CD209b and B220, was quantified as detailed in the Materials and Methods section. The CFSE-positive area of digital images was similar between GB2 wt and Cst-II mutant injected animals (A). No differences were observed in the CFSE-positive area within MOMA1 (B) or CD209b-positive cells (C). For each mouse, 6 pictures from 2 spleen sections were used for analysis (n = 4). Using Facs-analysis, the number of CFSE-positive metallophilic macrophages (Sialoadhesin+), red pulp macrophages (F4/80+), DC (CD11c+) and neutrophils (Ly-6G/C+) was determined per spleen (n = 3; D). Shown are means ± sd. * p<0.05; t-test with Welch's corrections.

CFSE-labelled GB2 was predominantly localised at the splenic marginal zone. This could be demonstrated by CFSE positive bacteria around the B-cell follicles at the border of the red and white pulp. Fluorescent bacteria could be identified in the spleens of animals sacrificed 15 min, 1 h or 3 h after i.v. injection. However, after 6 h, very few fluorescent particles could be observed. For (quantitative) colocalisation studies we therefore used the 1 h and 3 h time points. Immunofluorescent staining for sialoadhesin, which identifies marginal zone metallophilic macrophages, showed that both CFSE-labelled GB2 wt and Cst-II mutant were present in the sialoadhesin-expressing macrophages (Figure 3A and B). In the marginal zone, fluorescent bacteria were also observed in cells that were negative for sialoadhesin (Figure 4B, inset), suggesting uptake by marginal zone macrophages. Indeed, staining for CD209b (SIGNR1) on marginal zone macrophages, showed that GB2 was also scavenged by this cell type (Figure 3C and D).

The mean CFSE-positive area of digital images taken from the spleens of injected animals was used as an indication of the amount of bacteria present in each section. The percentage of the CFSE-positive area within metallophilic macrophages or marginal zone macrophages was used as a measurement of the co-localisation of the *C. jejuni* within these macrophage populations.

There was no difference in the CFSE-positive area between GB2 wt and Cst-II mutant-injected animals at either 1 h or 3 h indicating that the same quantity of sialylated and non-sialylated bacteria was deposited in the spleen following injection (Figure 4A). When the percentage of CFSE-positive area within the sialoadhesin or CD209b positive area was determined, no significant differences were observed between GB2 wt and Cst-II mutant-injected animals (Figure 4B and C).

To substantiate these findings to other phagocyte populations in the spleen, Facs-analysis was performed using markers specific for red pulp macrophages (F4/80), marginal zone metallophilic macrophages (MOMA1), DC (CD11c) and neutrophils (Ly-6G/C). The percentage of CFSE-positive splenocytes in GB2 wt injected animals (1.08±0.50%) was comparable to GB2 Cst-II mutant injected mice (1.01±0.20%). CFSE-labelled *C. jejuni* bacteria were detected in all phagocyte populations examined. No differences were observed in the number of CFSE-positive macrophages and neutrophils when comparing GB2 wt and Cst-II mutant-injected animals. However, a modest though significant increase was found in the number of DC that ingested the sialylated bacteria (p<0.05; Figure 4D).

### Sialylation of *C. jejuni* results in increased type I interferon production

We previously identified sialylation dependent differences in cytokine responses by myeloid cells to *C. jejuni* LOS *in vitro*. To determine if these effects were apparent after *in vivo C. jejuni* stimulation, RNA was isolated from the spleens of *C. jejuni* treated mice and the relative expression of a panel of cytokines was determined by real-time quantitative PCR.

**Figure 5:**
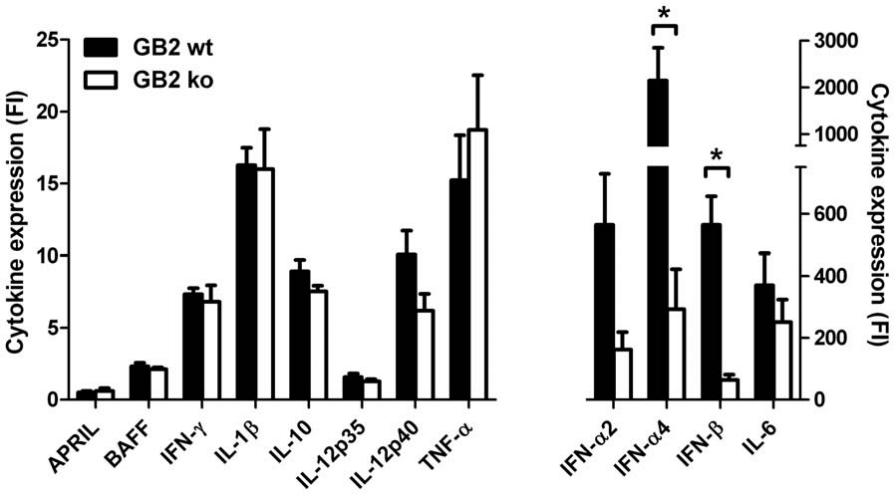
Increased type I interferon production in the spleens of GB2 wildtype injected animals. Mice were injected with PBS, GB2 wildtype or Cst-II mutant bacteria and spleens were isolated after 1 h. Spleen sections were homogenised for RNA extraction and cytokine expression was analysed by real-time PCR. Data is expressed as fold increase (mean ± sd; n = 4) compared to PBS-injected control animals. * p<0.05, Mann-Whitney U test.

With the exception of IFN-γ, all cytokines were expressed to a higher level at 1 h than at 3 h (data not shown). Highly upregulated cytokines, i.e. more than 25-fold compared to PBS-injected animals, included IFN-α2, IFN-α4, IFN-β and IL-6 (Figure 5). Both IFN-α4 and IFN-β expression were significantly higher in GB2 wt injected animals (fold increase of 2144±1410 and 564±183 respectively) compared to GB2 Cst-II mutant-injected animals (fold increase of 291±260 and 64±36; p<0.05; Mann-Whitney U test). IFN-α2 was also expressed to a higher level in GB2 wt-injected mice (fold increase of 508±309) as compared to mutant-injected mice (fold increase of 137±102), however this difference did not reach significance (p = 0.11; Mann-Whitney U test). The expression of other cytokines, amongst others TNF-α, IL-6, IL-10 and IL-1β, were not different between GB2 wt and GB2 mutant-injected animals.

The production of high levels of IFN-α2 and IFN-α4 after injection with *C. jejuni* may suggest involvement of pDC. To investigate whether pDC contributed to the production of cytokines especially type I IFN, we depleted pDC using the mAb 120G8 [20] prior to i.v. injection of *C. jejuni*. Administration of the depleting antibody significantly reduced the number of PDC1^+^ B220^+^ cells in the CD11c^+^ CD11b^−^ population, demonstrating that the large majority of splenic pDC were depleted (Figure S4A). Induction of type I IFN as well as several other cytokines (i.e., IL-6 and TNF-α) was observed in spleens of pDC-depleted animals after injection with GB2 wt (Figure S4B). There was no difference between pDC-depleted animals and isotype control treated animals, indicating that the production of cytokines, including type I IFN was pDC-independent.

To study whether the increased expression of type I IFN could also be detected systemically, we measured cytokines in serum samples collected either at 3 h or at 16 h after *C. jejuni* injection. No IFN-α or IFN-β could be detected in serum samples of mice injected with wildtype of Cst-II mutant *C. jejuni* strains.

In summary, although injection of both sialylated and unsialylated *C. jejuni* induced the production of several pro-inflammatory cytokines in the spleen, sialylated *C. jejuni* was significantly more effective in inducing a type I IFN response.

## Discussion

LOS sialylation is a crucial factor in the pathogenesis of GBS as the sialic acid residue is a major determinant of the structural similarity with human nerve gangliosides. In addition, sialylation is associated with more severe gastroenteritis and higher levels of *C. jejuni*-specific IgM [12] indicating a stimulatory effect on host immune responses. As myeloid cells drive innate and adaptive immune responses, we hypothesised that sialylation of LOS affects functional responses of myeloid cells, through interaction with sialic acid specific receptors. Using a combination of in vitro and in vivo models, we sought to investigate if sialylation influences the responses of these cells in both their capacity to phagocytose *C. jejuni* and their cytokine responses to purified LOS. Our results show that sialylation of *C. jejuni* increases phagocytosis by BM-MΦ *in vitro*, an effect most likely mediated by sialoadhesin, whereas *in vivo* studies indicated a preferential uptake of sialylated *C. jejuni* by splenic DC. The production of cytokines by myeloid cells was clearly increased by sialylated *C. jejuni*. This effect was not only observed *in vitro* by both macrophages and DC, but also *in vivo*, where type I interferons were identified as major discriminators between sialylated *C. jejuni* strains and their unsialylated counterpart. The findings in this study will be crucial for further understanding of the increased pathogenicity of sialylated *C. jejuni* strains and its mechanisms to induce cross-reactive humoral responses leading to GBS.

The functional implications of *C. jejuni* sialylation on myeloid cells are not fully known. Following up on the recent findings by Bax et al. [21] demonstrating that sialylation of *C. jejuni* LOS modulates Th1/Th2 skewing by human DC, we further elucidate the role of microbial sialylation on myeloid cell responses. While sialylation of *C. jejuni* increased phagocytosis by cultured BM-MΦ it did not increase phagocytosis by BM-DC. Sialoadhesin is expressed on BM-MΦ but not BM-DC, suggesting that *in vitro* sialoadhesin might mediate increased phagocytosis. In support of this, a sialoadhesin-mediated phenomenon has previously been reported with meningococci [18]. However, although the increased phagocytosis of sialylated *C. jejuni in vitro* is likely mediated by sialoadhesin, we did not find that *C. jejuni* was preferentially taken up by sialoadhesin-positive marginal zone metallophilic macrophages in the spleen. This discrepancy may be explained by the absence of serum factors like complement and immunoglobulin during the *in vitro* studies. Receptors recognising complement are highly expressed by macrophage populations in the marginal zone, explaining the preferential location of both strains of *C. jejuni*. It must also be noted that in order to facilitate tracking of *C. jejuni*, we made use of heat-inactivated bacteria, which may differ substantially from live bacteria. For example, live bacteria are able to change the composition of their outer core depending on growth rate and environmental factors i.e. temperature and availability of nutrients. In addition, the i.v. injection of mice with heat-killed *C. jejuni* does not fully represent the natural route of infection via the intestinal mucosa. Nevertheless, our data are relevant for understanding the pathogenesis of *C. jejuni*. As a consequence of bacterial translocation across epithelial barriers, bacteria may enter the blood stream relatively early during disease. In mice infected i.g. with *C. jejuni*, bacteraemia is present as early as 10 min after inoculation and resolves within 24 h [22], [23]. Transient bacteraemia is also thought to occur in humans, especially in patients with high fever [24]. Skirrow et al. [25] reported that *C. jejuni* bacteraemia occurred in 0.3–5.9 per 1000 intestinal infections. The incidence increased with age and was twice as frequent in men as in women. Most probably the frequency of bacteraemia is underestimated, as it occurs early in disease when blood cultures are rarely taken [24]. The crucial role of the spleen in the recovery from *C. jejuni* bacteraemia is exemplified by a case report describing fatal outcome after *C. jejuni* infection in a splenectomised patient [26]. Further studies to investigate the role of myeloid cells in immunity to sialylated *C. jejuni* should take these bacterial changes into account.

In agreement with our previous observations on LOS potency in human DC [13], we demonstrated that sialylation of *C. jejuni* LOS dramatically enhanced the *in vitro* production of cytokines, both by macrophages and DC. We also found that both *C. jejuni* strains induced a robust cytokine response *in vivo*, including type I IFN, IL-10, IL-6, TNF-α and IL-12, in the spleen 1 h after injection. Strikingly, the only difference between the strains was found in the production of type I interferon which was higher in mice injected with sialylated *C. jejuni*. This effect is most likely attributed to increased signalling of sialylated LOS via TLR4 [13]. Our finding that more CD11+ DC, which are known to express TLR4, were phagocytosing GB2 wt may explain the type I IFN signature in response to sialylated bacteria. TLR4 activation leads to both MyD88-dependent and MyD88-independent signal transduction, the latter being responsible for the induction of IFN-β [22]. In another study, *C. jejuni* has indeed been shown to induce IFN-β in mouse BM-DC in a TLR-4 and TIR-domain-containing adapter-inducing interferon-β (TRIF)-dependent manner [19].

TLR2 in conjunction with TLR1 and TLR6 may also play a role in the production of cytokines in response to *C. jejuni* [27]. Our observation that IL-10, IL-6, TNF-α and other cytokines were not significantly different between sialylated and non-sialylated strains may be explained by the additional signalling of *C. jejuni* via TLR2, thus decreasing the relative contribution of TLR4 signalling for the production of these cytokines. Alternatively, our inability to show a difference in IL-6 and IL-10 levels may represent the lack of specificity in our assay, which looks at whole spleen tissue. This does not take into account the microenvironment around individual antigen presenting cells, such as DC, in which differences in IL-6 and IL-10, consistent with our *in vitro* results, may exist and influence nearby immune effector cells.

The increased induction of IFN-α and IFN-β by sialylated *C. jejuni* may play a role in the subsequent production of anti-ganglioside antibodies that cause GBS. Type I interferons directly act on B cells by decreasing the threshold of BCR activation [28], promoting isotype switching and increasing immunoglobulin production [17]. Its crucial role in the immune response is further illustrated by the fact that the antibody-augmenting effect of complete Freund's adjuvant can be largely attributed to the production of type I IFN [29]. Because of its important role in humoral immune responses, we speculate that the increased levels of specific IgM responses in patients infected with sialylated *C. jejuni* [12] may be due to the selective increase in type I IFN by the sialylated pathogen. In susceptible persons, this increase could subsequently facilitate the activation of ganglioside cross-reactive B cells. In the study of Mortensen et al, no difference in IgG levels was observed between subjects infected with sialylated and unsialylated *C. jejuni*. It is possible that due to local production type I IFN only affects newly activated B cells and has little influence on long-lived plasma cells, which contribute the most to peripheral IgG levels.

Importantly, treatment of hepatitis or multiple sclerosis patients with IFN-α or IFN-β respectively can lead to the development of autoimmune neuropathies [30], [31]. Although a systematic epidemiologic study on this matter is lacking, it appears that type I IFN-induced neuropathy is not a general effect but rather occurs in a small minority of cases. Not only is it necessary that the B cells with ganglioside cross-reactivity are present in the subject, the B cells also need to be activated through the BCR, as type I IFN only promotes humoral responses in a BCR-dependent manner [28]. This requires an infection with pathogens expressing ganglioside mimics, such as *C. jejuni* or *Haemophilus influenzae* [32].

In conclusion, our data indicate that sialylation of *C. jejuni* results in increased phagocytosis by murine macrophage *in vitro* and increased scavenging of *C. jejuni* by splenic DC *in vivo*. Sialylated *C. jejuni* is more effective at inducing cytokine responses by myeloid cells, particularly type I interferons. Future studies must address to what extent these cytokine responses contribute to exacerbated disease and development of post-infectious complications such as GBS by sialylated *C. jejuni* strains.

## Materials and Methods

### Culture and fluorescent labelling of *C. jejuni*

*C. jejuni* strains GB2 and GB11 were isolated from patients with GBS, and the corresponding Cst-II mutant strains, producing LOS lacking sialic acid were described previously [33]. Bacteria were cultured on blood agar plates incubated at 37°C under microaerophilic conditions and harvested after 48–72 h. To obtain fluorescently labelled bacteria, cells were washed with PBS and resuspended to an OD_600_ of 1.0. An equal volume of 2 µM CFDA-SE in PBS was added and cells were incubated for 30 min at 37°C and subsequently heat-inactivated for 1 h at 65°C. Bacteria were washed twice with PBS and stored at −80°C in PBS/10% glycerol until use.

### Phagocytosis of *C. jejuni* by BM-MΦ and BM-DC

For BM-MΦ, C57BL/6 bone marrow cells (0.5×10^6^/ml) were cultured in 90 mm Petri dishes (Sterlin, UK) in RPMI supplemented with 20% L-929 supernatant (containing M-CSF) and incubated at 37°C in 5% CO_2_. Fresh medium was added on day 4. Adherent cells were harvested on day 7 using a cell scraper and ice cold PBS. Purity was assessed by flow cytometry and was typically >90% F4/80^+^. BM-DC were grown using a similar protocol only the RPMI was supplemented with 10% X-63 supernatant (containing GM-CSF) with 10 ml of fresh RPMI/10% X-63 added on day 4. Non-adherent cells were harvested on day 8. Purity was assessed by flow cytometry and was typically >70% CD11c^+^. For the phagocytosis assays, RPMI medium supplemented with Ultra-low IgG foetal bovine serum (Invitrogen, UK) was used to exclude the possibility of bovine IgG affecting phagocytosis. Macrophages or DC (1×10^6^/ml) were incubated at 37°C with CFSE-labelled GB11 wt or mutant bacteria at a ratio of 10 or 20∶1. As a control, cells were incubated in the presence of 5 mM NaN_3_ at 4°C. After 0, 15, 30 or 60 minutes, cells were washed with ice cold PBS with 5 mM NaN_3_, stained with anti-mouse F4/80 biotin or anti-mouse CD11c biotin and streptavidin APC and measured on a Facs Calibur. For analysis, F4/80^+^ or CD11c^+^ cells were gated and the mean fluorescence intensity (MFI) of these cells (representing phagocytosed bacteria) was determined and divided by the MFI of the 4°C, NaN_3_ cultured controls for each time point (n = 3). The residual sample following Facs analysis was centrifuged onto plates and visualised using an Apotome microscope to confirm the intracellular location of the bacteria.

### Stimulation of myeloid cells by *C. jejuni* LOS

LOS was purified from *C. jejuni* using previously published protocols [13]. BM-DC and BM-MΦ were generated as above and cultured at 1×10^5^ cells per well in 96 well flat-bottom plates (Nunc) with the appropriate concentration of LOS. The supernatants were harvested after overnight culture at 37°C with 5% CO2 and analysed using an IL-6 ELISA kit from R&D and an OptEIA IL-10 ELISA kit (BD Biosciences, UK). Supernatants isolated after 4 h stimulation were used to determine IFN-β production using ELISA (PBL InterferonSource, Piscataway, NJ). For C3H/HeJ and C3H/HeN experiments, mice were purchased from Harlan UK and BM-DC were generated as above.

### Intravenous challenge of mice with *C. jejuni*

C57BL/6 mice (8–12 weeks old) were bred and housed under standard laboratory conditions at the University of Glasgow (Glasgow, Scotland). All experiments were performed under the UK Home Office License.

A 200-µl suspension containing 10^8^ bacteria (OD_600_ of 0.6), or PBS as a negative control, was injected into the tail vein. Mice were sacrificed after 15 min, 1 h or 3 h using CO_2_. Organs were snap-frozen in OCT using liquid nitrogen. Alternatively, the spleen was immediately passed through a 100-µm cell strainer and splenocytes were kept on ice in PBS/2 mM EDTA for Facs-analysis. pDC depletion was accomplished by administrating 250 µg 120G8 or isotype control antibody/mouse i.p. 24 h and 48 h before injection of bacteria.

### Facs-analysis

Splenocytes were fixed with 2% paraformaldehyde in PBS on ice for 30 min, washed and incubated with 5 µg/ml Fc-block (BD) for 30 min. Primary antibodies were added to the cells (6×10^5^ cells/staining): biotinylated anti-F4/80 (10 µg/ml; clone BM8; Biolegend), biotinylated anti-CD11c (10 µg/ml; clone HL3; BD), biotinylated IgG1 isotype control (10 µg/ml; BD), APC-conjugated anti-CD19 (2 µg/ml; clone 1D3; BD), APC-conjugated anti-Ly-6G/C (4 µg/ml; clone RB6-8C5; eBiosience, Hatfield, UK), APC-conjugated isotype control (4 µg/ml; eBiosience) or MOMA1 (hybridoma supernatant, kindly provided by Dr. P. Leenen). Secondary conjugates included biotinylated anti-rat IgG2a (5 µg/ml; clone MARG2a-1; Serotec) and APC-conjugated streptavidin (2 µg/ml; BD). pDC staining was performed using Alexa Fluor 488-conjugated anti-CD11b, PE/Cy7-conjugated anti-CD11c (Biolegend, San Diego, CA), PerCP-eFluor710-conjugated anti-PDCA1 (eBioscience) and PE-conjugated anti-B220 (BD). Cells were measured on a Facs Calibur or Facs Canto II HTS and data was analysed using FlowJo software (version 8.8.6).

### Immunofluorescence

Frozen sections (6 µm) were fixed for 10 min with acetone, air-dried and washed with PBS/0.05% Tween. Aspecific binding was blocked with 10% normal goat serum and primary antibodies in PBS 1% BSA were allowed to bind for 1 h at room temperature (20°C). Primary antibodies included MOMA-1 and ER-TR9 (anti-CD209b) (both hybridoma supernatants, kindly provided by Dr. P. Leenen). Sections were subsequently incubated with 5 µg/ml Tetramethyl Rhodamine Isothiocyanate (TRITC)-labelled goat anti-rat IgM or IgG (Southern Biotech Associates) and stained with 6.67 µg/ml Alexa 647-labelled anti-B220 (clone RA3-6B2; Biolegend). To prevent binding to anti-rat IgG-TRITC, sections were blocked with 1% rat serum prior to incubating with anti-B220. Sections were mounted with Citifluor anti-fadent solution (Citifluor Ltd., Leicester, UK).

### Quantification of fluorescently labelled *C. jejuni* in spleen sections

Six digital images (three per section) of splenic follicles were taken from each animal using an Apotome Zeiss microscope. Digital images were quantified using Fiji software. The total CFSE-positive area (above threshold level) of all images was calculated. In addition, the percentage of area containing CFSE was determined within the sialoadhesin or CD209b-positive area. Sections of PBS-injected animals and sections incubated with secondary antibodies only were used to determine threshold levels.

### Real-time quantitative PCR

Three to six 30-µm sections were cut from snap-frozen spleens and kept on dry ice until further processing. Tissue was homogenised in 500 µl RNA-Bee solution (Tel-Test, Friendswood, TX, USA), 100 µl chloroform was added and samples were thoroughly mixed. Following centrifugation, the aqueous phase was collected and RNA was further purified using the GenElute mammalian total RNA isolation kit (Sigma-Aldrich). Using 1 µg of total RNA template, cDNA was prepared using SuperScript II reverse transcriptase (Invitrogen) and oligo(dT) and random hexamer primers. Cytokine and GAPDH RNA levels were measured by real-time quantitative PCR analysis using the ABI PRISM 7700 sequence detection system (Applied Biosystems). PCR primers were spanning at least one intron/exon boundary. Sequences for the primers and reference numbers for probes (Universal Probe Library; Roche Applied Science) are listed in Table S1. RNA levels were calculated relative to amounts found in a standard sample, and cytokine levels were corrected for GAPDH RNA levels to normalize for RNA input.

### Serum cytokine ELISA

IFN-α and IFN-β levels were measured by ELISA (PBL) in serum samples of mice sacrificed 3 h or 16 h after GB2 injection. Assays were performed according to the manufacturer's guidelines.

### Statistical analysis

GraphPad Prism was used for all statistical analyses; t-test, t-test with Welch's corrections or Mann Whitney *U* as appropriate. Data are represented as mean ± SD or SEM as indicated. *P*≤0.05 was considered statistically significant and all tests were two sided.

## Supporting Information

Figure S1 **CFSE-labelling of wildtype and Cst-II knockout GB2 and GB11 results in comparable fluorescence intensities.** GB2 or GB11 wt and Cst-II mutant bacteria were either left untreated (filled histograms) or were incubated with 1 µM CFSE for 30 min at 37°C, resulting in equal MFI.
(TIF)

Figure S2 **Incubation of** ***C. jejuni*** **with BM-MΦ leads to uptake into an intracellular location.** BM-MΦ were incubated with CFSE-labelled wt GB11 (green) at 37°C or 4°C (with NaN_3_). A cytospin was made after 60 minutes and internalisation of *C. jejuni* was visualised using fluorescence microscopy, following mounting with DAPI containing media. Shown is a z-stack of GB11 wt *C. jejuni* (GB11 ko gave similar results). Inset in (C) shows clearly identifiable bacteria not present in the top (A) or bottom (D) image, confirming phagocytosis.
(TIF)

Figure S3 **Sialoadhesin is expressed on BM-MΦ and is upregulated upon** ***C. jejuni*** **LOS stimulation.** Cells were stimulated overnight with GB11 wt or Cst-II mutant LOS and sialoadhesin expression was examined by flow cytometry. Sialoadhesin was expressed on unstimulated BM-MΦ (A; dotted line) and was upregulated after stimulation with GB11 LOS (B, and solid line in A). Filled histograms represent background fluorescence when incubated with the secondary antibody alone. BM-DC only express low levels of sialoadhesin and do not upregulate sialoadhesin in response to GB11 LOS (C).
(TIF)

Figure S4 **Type I interferon expression in response to** ***C. jejuni*** **is pDC-independent.** Mice were pretreated with 250 µg 120G8 antibody or an isotype control antibody, 24 h and 48 h before injection with 10^8^ GB2 wt bacteria. The majority of the pDC were depleted as indicated by a strong reduction in the percentage of PDCA1^+^ B220^+^ cells within the CD11b^−^ CD11c^+^ gate (A). Cytokine expression was determined in the spleen by qPCR and revealed no significant differences between 120G8 and control antibody treated mice (B).
(TIF)

Table S1 **Primer sequences and probe numbers used for real-time quantitative PCR.**
(DOCX)
