# Supplementary figures and images for: Sialylation of Campylobacter jejuni Lipo-Oligosaccharides: Impact on Phagocytosis and Cytokine Production in Mice
Source: PLoS One. 2012 Mar 28;7(3):e34416. doi: 10.1371/journal.pone.0034416 (PMC3314637; doi:10.1371/journal.pone.0034416)

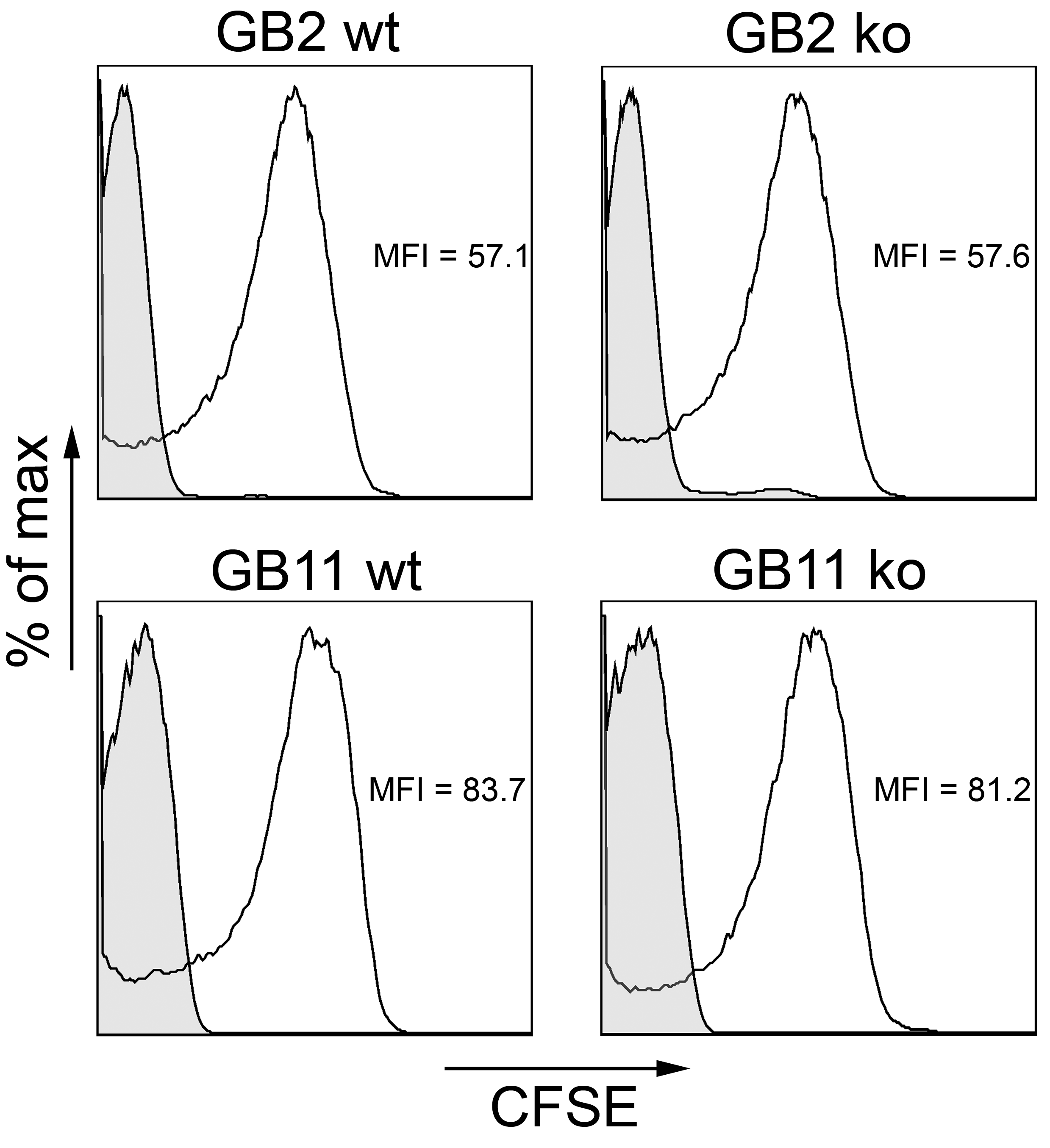

Supplement: Figure S1 — CFSE-labelling of wildtype and Cst-II knockout GB2 and GB11 results in comparable fluorescence intensities. GB2 or GB11 wt and Cst-II mutant bacteria were either left untreated (filled histograms) or were incubated with 1 µM CFSE for 30 min at 37°C, resulting in equal MFI. (TIF) [file pone.0034416.s001.tif]

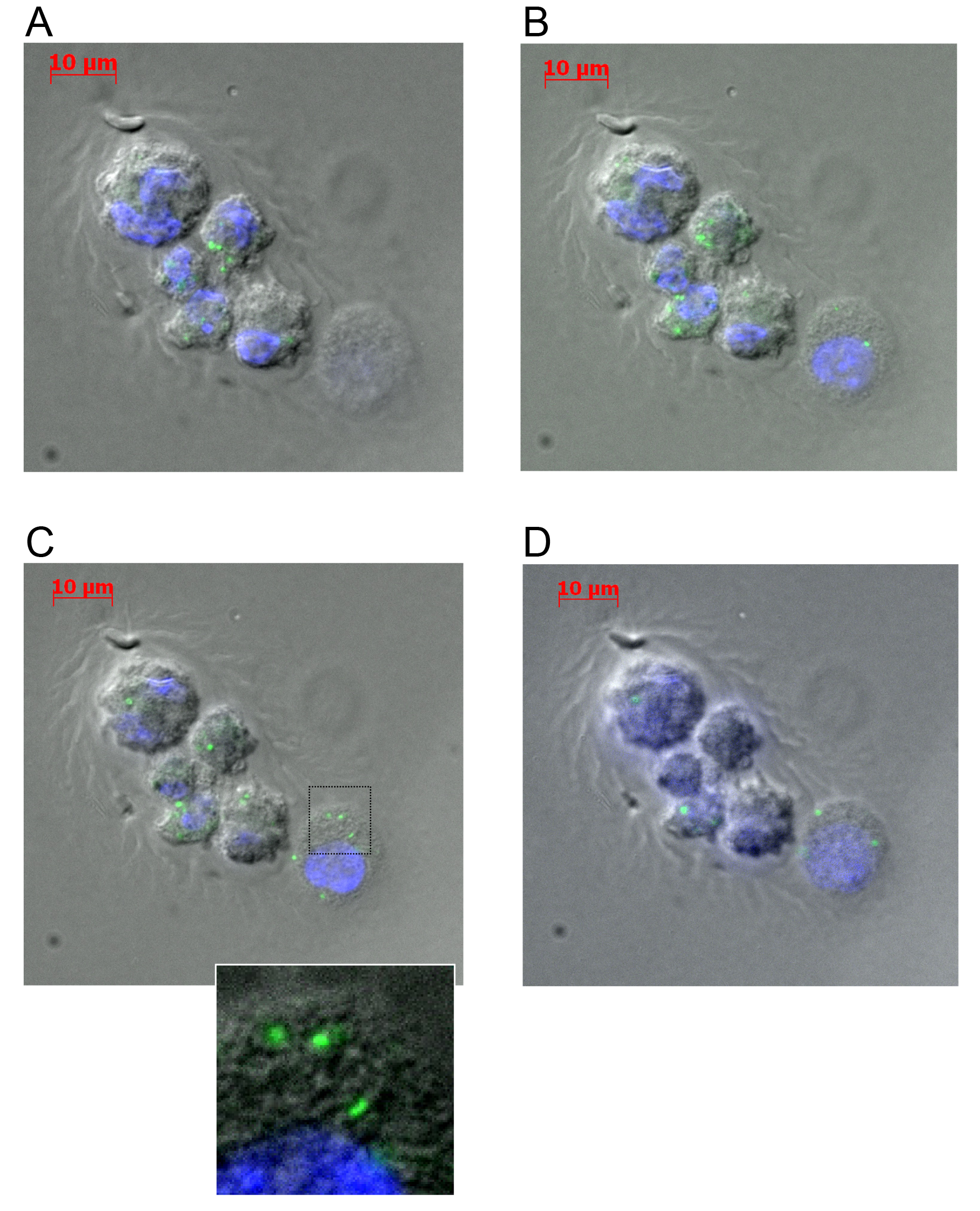

Supplement: Figure S2 — Incubation of C. jejuni with BM-MΦ leads to uptake into an intracellular location. BM-MΦ were incubated with CFSE-labelled wt GB11 (green) at 37°C or 4°C (with NaN3). A cytospin was made after 60 minutes and internalisation of C. jejuni was visualised using fluorescence microscopy, following mounting with DAPI containing media. Shown is a z-stack of GB11 wt C. jejuni (GB11 ko gave similar results). Inset in (C) shows clearly identifiable bacteria not present in the top (A) or bottom (D) image, confirming phagocytosis. (TIF) [file pone.0034416.s002.tif]

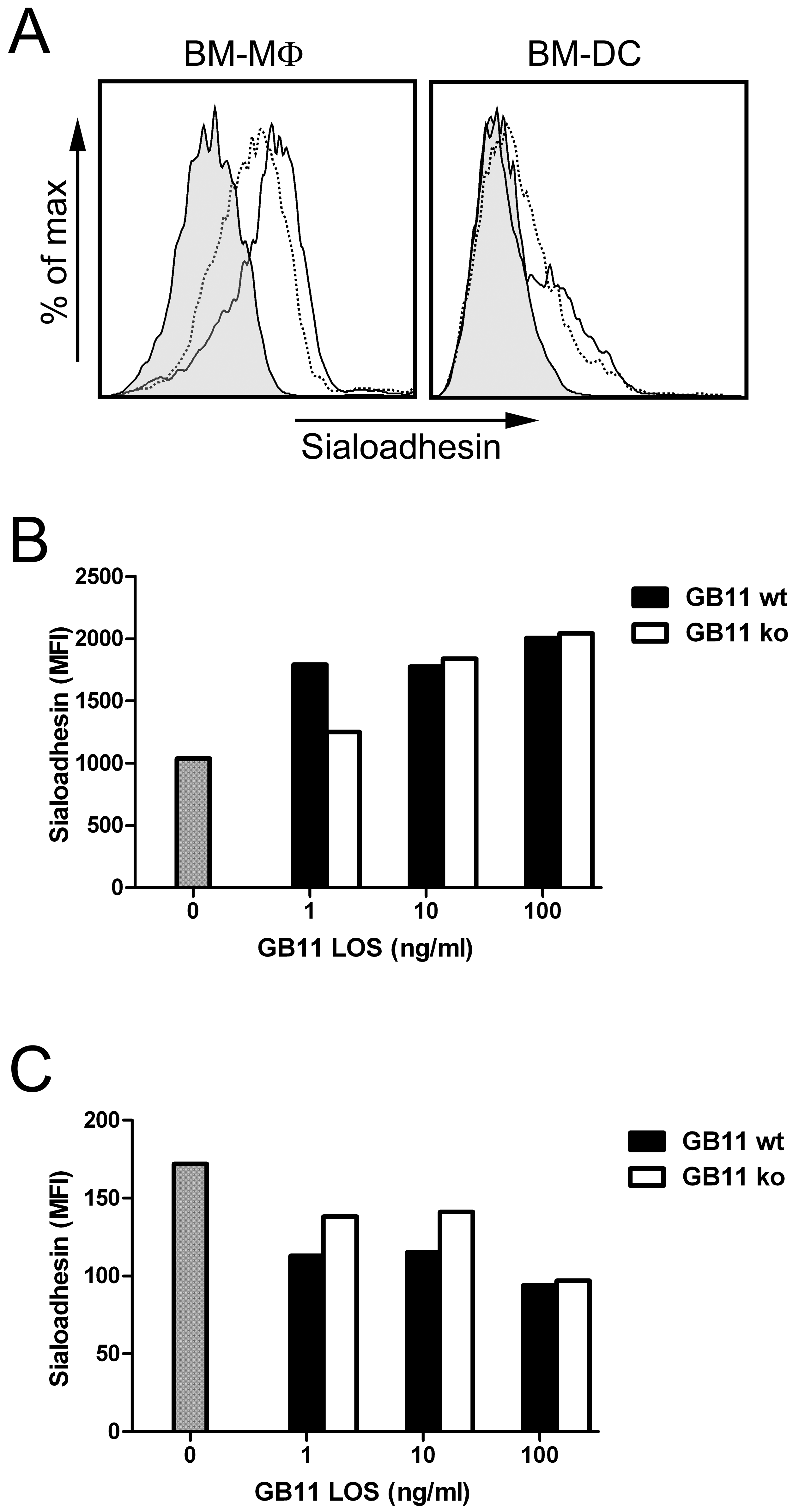

Supplement: Figure S3 — Sialoadhesin is expressed on BM-MΦ and is upregulated upon C. jejuni LOS stimulation. Cells were stimulated overnight with GB11 wt or Cst-II mutant LOS and sialoadhesin expression was examined by flow cytometry. Sialoadhesin was expressed on unstimulated BM-MΦ (A; dotted line) and was upregulated after stimulation with GB11 LOS (B, and solid line in A). Filled histograms represent background fluorescence when incubated with the secondary antibody alone. BM-DC only express low levels of sialoadhesin and do not upregulate sialoadhesin in response to GB11 LOS (C). (TIF) [file pone.0034416.s003.tif]

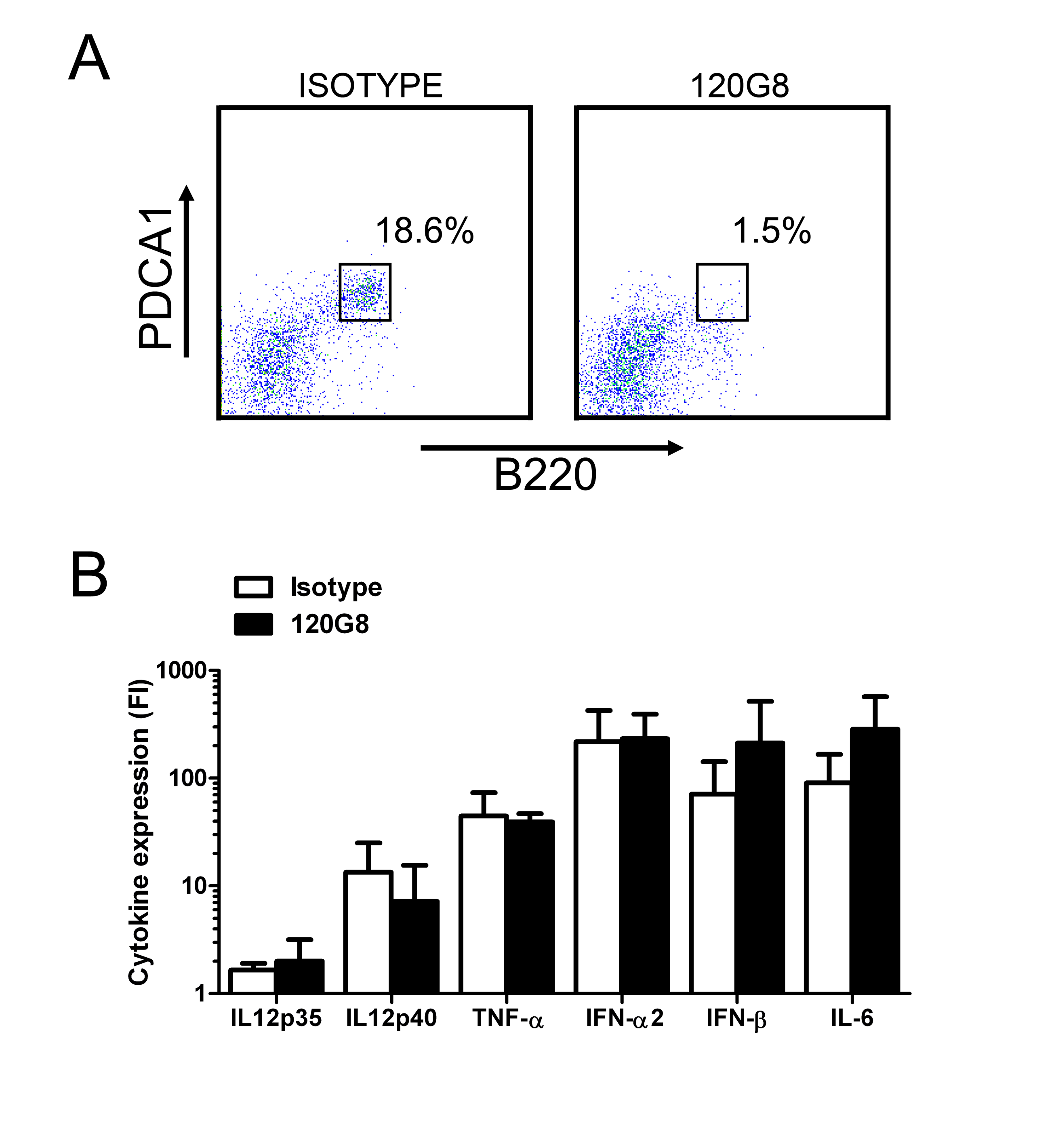

Supplement: Figure S4 — Type I interferon expression in response to C. jejuni is pDC-independent. Mice were pretreated with 250 µg 120G8 antibody or an isotype control antibody, 24 h and 48 h before injection with 108 GB2 wt bacteria. The majority of the pDC were depleted as indicated by a strong reduction in the percentage of PDCA1+ B220+ cells within the CD11b− CD11c+ gate (A). Cytokine expression was determined in the spleen by qPCR and revealed no significant differences between 120G8 and control antibody treated mice (B). (TIF) [file pone.0034416.s004.tif]
