# Supplementary material for: Sialylation of Campylobacter jejuni Lipo-Oligosaccharides: Impact on Phagocytosis and Cytokine Production in Mice
Source: PLoS One. 2012 Mar 28;7(3):e34416. doi: 10.1371/journal.pone.0034416 (PMC3314637; doi:10.1371/journal.pone.0034416)
Supplement: Table S1 — Primer sequences and probe numbers used for real-time quantitative PCR. (DOCX) [file pone.0034416.s005.docx]

**Table S1. Primer sequences and probe numbers used for real-time quantitative PCR.**

| **Molecule** | **Forward primer** | **Reverse primer** | **Probe no.*** |
| --- | --- | --- | --- |
| **APRIL** | GGTGGTATCTCGGGAAGGAC | CCCCTTGATGTAAATGAAAGACA | 7 |
| **BAFF** | AACACTGCCCAACAATTCCT | TGCAAGCTGAATCTCATCTCC | 11 |
| **GAPDH** | AGCTTGTCATCAACGGGAAG | TTTGATGTTAGTGGGGTCTCG | 9 |
| **IFN-α2** | Applied Biosystems: Mm_00833961_S1 | | |
| **IFN-α4** | Applied Biosystems: Mm_00833969_S1 | | |
| **IFN-β** | Applied Biosystems: Mm_00439546_S1 | | |
| **IFN-γ** | GCAAAAGGATGGTGACATGA | TTCAAGACTTCAAAGAGTCTGAGG | 21 |
| **IL-1β** | CAAAAGATGAAGGGCTGCTT | GAAGCTGGATGCTCTCATCA | 26 |
| **IL-6** | GATGGATGCTACCAAACTGGAT | CCAGGTAGCTATGGTACTCCAGA | 6 |
| **IL-10** | GCTCCTAGAGCTGCGGACT | TGTTGTCCAGCTGGTCCTTT | 41 |
| **IL-12p35** | GAGACTTCTTCCACAACAAGAGG | CAGGGTCATCATCAAAGACG | 27 |
| **IL-12p40** | TGGACTGGACTCCCGATG | CATCTTCTTCAGGCGTGTCA | 80 |
| **TNF-α** | CCACGTCGTAGCAAACCAC | TTTGAGATCCATGCCGTTG | 25 |

* Universal probe library (Roche)
